# Short-term effects of community-based marine reserves on green abalone, as revealed by population studies

**DOI:** 10.1038/s41598-023-50316-9

**Published:** 2024-01-10

**Authors:** Jeremie Bauer, Jaime Segovia-Rendón, Julio Lorda, Alicia Abadía-Cardoso, Luis Malpica-Cruz, Patricia Alvarado-Graef, Ricardo Searcy-Bernal, Leonardo Vázquez-Vera, Rodrigo Beas-Luna

**Affiliations:** 1 Facultad de Ciencias Marinas, Universidad Autónoma de Baja California, Carretera Ensenada-Tijuana 3917, 22860 Ensenada, Baja California Mexico; 2 Departamento de Biotecnología Marina, Centro de Investigación y Estudios Superiores de Ensenada, Carretera Ensenada-Tijuana 3918, 22860 Ensenada, Baja California Mexico; 3 Proyectos y Servicios Marinos (PROSEMAR), Colinas de Ensenada 209, 22760 Ensenada, Baja California Mexico; 4 Facultad de Ciencias, UABC, Carretera Ensenada-Tijuana 3917, 22860 Ensenada, Baja California Mexico; 5 Tijuana River National Estuarine Research Reserve, 301 Caspian Way, Imperial Beach, CA 91932 USA; 6 Instituto de Investigaciones Oceanológicas, UABC, Carretera Ensenada-Tijuana 3917, 22860 Ensenada, Baja California Mexico; 7 ECOCIMATI, A.C., Av. Del Puerto 2270 Colonia Hidalgo, 22880 Ensenada, Baja California Mexico; 8 Universidad Autónoma de Baja California Sur (UABCS), Carretera al Sur KM 5.5, 23080 La Paz, Baja California Sur Mexico

**Keywords:** Conservation biology, Population dynamics

## Abstract

Marine reserves (MRs) are implemented worldwide to protect, restore, and manage marine ecosystems and species. However, it is important to document the positive effects those marine reserves have on slow-growth, temperate invertebrates such as abalone. Abalone, *Haliotis* spp., are marine gastropods of high economic value extracted worldwide for decades, which has led to fisheries-driven population decreases. In this work, we focused on a case study and assessed the short-term (1–2 years) effects of marine reserves established and managed by a local fishing cooperative at Guadalupe Island, Mexico. We evaluated the population status of green abalone, *H. fulgens*, by conducting (1) an assessment of the green abalone population around Guadalupe Island through subtidal monitoring and (2) an evaluation of the effect of two recently established marine reserves on population parameters such as the increase in density (individuals·m^2^), biomass, number of aggregated abalone, egg production, and proportion of individuals bigger than 150 mm (minimum harvest size) compared to fished areas. To assess the population around Guadalupe Island, we surveyed 11,160 m^2^ during 2020 and 2021. We recorded 2327 green abalones with a mean ± SE shell length of 135.978 ± 0.83 mm and a mean density of 0.21 ± 0.02 individuals·m^2^. All variables were statistically higher at the MRs except for shell length in 2021. In this work, we report for the first time the green abalone population status at Guadalupe Island and a positive short-term biological response to community-based marine reserves. This study suggests that a network of MRs combined with good management could help abalone populations in the short term in Guadalupe Island, potentially leading to more sustainable fishing practices and social-ecological resilience.

## Introduction

Marine protected areas or marine reserves (MR) have the potential to rebuild depleted populations^1^, increase fisheries yields through adult^2^ and larvae spillover^3,4^, protect biodiversity^5^, empower local communities^6^, and assure the continuing flow of ecosystem services^7^. In addition, establishing MRs is a well-documented approach for reducing local disturbance of selective fishing and stock collapses from overexploitation^8–10^. It is crucial to document the effects of a MR on target species or the ecological community, specifically focusing on the time required to observe these effects to understand the associated benefits.

Marine reserves can cause biological responses (e.g., winners and losers responding to survival, growth, recruitment, etc.), but how quickly these effects occur in different ecoregions, communities, and species is unclear. Species and communities can take several different trajectories after establishing a MR, depending on various biological, environmental, location, and social factors^5,11,12^. For example, MRs can increase levels of density and biomass within 1–3 years on average after closure to fishery^13^. However, this remains uncertain for slow-growing temperate invertebrates such as abalone.

The expected short-term effects of marine reserves on slow-growing species compared to faster-growing species are fundamentally different. Slow-growing species often face challenges in achieving rapid recovery due to their extended generation times^14^, limited reproductive rates, and vulnerability to overfishing^15^. In contrast, faster-growing species tend to rebound more quickly from population declines^5^. However, some evidence suggests that MRs could have short-term positive effects on the recovery of slow-growing species due to immigration or population distribution changes, for example, from deeper areas where fishing pressure is lower^16^. These differences underscore the importance of considering the different species' responses, given their distinct ecological dynamics and life history traits, when evaluating the effectiveness of MRs.

Worldwide, different types of MRs exist depending on the level of protection provided to the ecosystem^17^. Mexico has a long history of protecting the marine environment, with MRs totaling around 700,000 km^2^ in the present^18^. One example of success are community-based marine conservation initiatives establishing no-take MRs within fishing concession zones^19^. Unlike top-down MRs—those established and administered by government agencies—community-based MRs are inherently connected to the community and their well-being, thus fostering social-ecological conservation benefits^20^.

In the Baja California Peninsula, community-based no-take MRs tailored to protect and enhance the populations of fished species have been traditionally used^21^. In 2006, Natividad Island was the first example where these MRs were formalized, incorporating citizen science and results assessment^22^. Within these MRs, pink abalone (*Haliotis corrugata*) populations have not only maintained their stability in terms of size and egg production but have also demonstrated greater resilience and faster recovery following a severe mass mortality event^23^.

Abalone is one of the most valuable commercial marine gastropods and a classic example of overfishing^24^. Globally, wild abalone fisheries landings have decreased significantly in the past decades, from 20,000 metric tons (mt) in the 1970s to only about 4500 mt in 2021^25^. Fishing pressure, combined with disease outbreaks and environmental factors, has depleted wild abalone populations^26^. Moreover, as relatively sedentary broadcast spawners, abalones are prone to Allee effects, with low densities leading to low breeding success^27^. A prime example is the white abalone, *H. sorenseni*, which faces extinction in California, USA, even after 25 years of fishery closure^28^. Due to low abundances, its populations cannot recover independently without human intervention. Hence, it is crucial to continue implementing management and conservation actions such as MRs to recover these economically and culturally important organisms.

In Mexico, the abalone artisanal fishery occurs on the Pacific coast of the Baja California Peninsula, and from 1955 to 1975, it supported mean catches of 3000 mt per year, with five abalone species^29^. Currently, the abalone fishery produces only about 322 mt^25^ consisting of two species, 96% green (*H. fulgens*) and the remaining 4% pink (*H. corrugata*) abalone. This fishery historically served as the primary source of income for coastal communities in Baja California. However, this has changed, and today, the red lobster *Panulirus interruptus* fishery has taken the lead in productivity^21^. This shift can be attributed to a decline in wild abalone populations, largely due to the adverse effects of overfishing and environmental impacts. Notably, one of the few cooperatives where abalone still holds its central role is “Abuloneros y Langosteros”, established on Guadalupe Island during the 1980s. Consequently, they are actively working on developing fishery management and conservation measures aimed at safeguarding wild abalone populations.

These management strategies encompass rotational fishing conducted within designated polygons throughout the 5-month fishing season (Mexican Zone 1 in 2021; fishery: February 1st–June 30th; complete closure: July 1st–January 31st). Based on local ecological knowledge, the cooperative manages each polygon depending on the fishing pressure it can support through the season, aiming to not go under abalone densities of 0.2 individuals·m^2^. This approach has been consistently employed and is deeply rooted in the cooperative's practical knowledge acquired over the years. Additionally, the cooperative adheres to specific regulatory measures, including a minimum harvest size of 150 mm for *H. fulgens* and a quota limiting the number of organisms harvested per fishing day.

Furthermore, a significant recent development within the cooperative's conservation efforts is the establishment of two community-based MRs. The first MR was implemented in 2019, followed by the second in 2020. It is important to note that these MRs are classified as no-take, which means that any form of extraction activity is strictly prohibited within their boundaries. Importantly, the enforcement of these no-take MRs is carried out by the cooperative's members, emphasizing their commitment to sustainable fishing practices and marine conservation. Despite the significance of the Guadalupe Island green abalone fishery, there are no population studies published.

Even if the short-term effects of MRs on size have been documented^30^, abalone are known for their slow growth, which means that observing a noticeable change in shell length within 1–2 years is generally considered a relatively short time frame. However, it is interesting to assess the short-term effects the fishery closure has on other biological parameters. The objectives of this study were: (1) to assess the green abalone population around Guadalupe Island through subtidal monitoring and (2) to evaluate the effect of the newly established MRs on population parameters such as density, biomass, number of aggregated abalone, potential egg production, and proportion of individuals bigger than 150 mm (minimum harvest size) compared to fished areas.

## Materials and methods

### Study site

**Figure 1:**
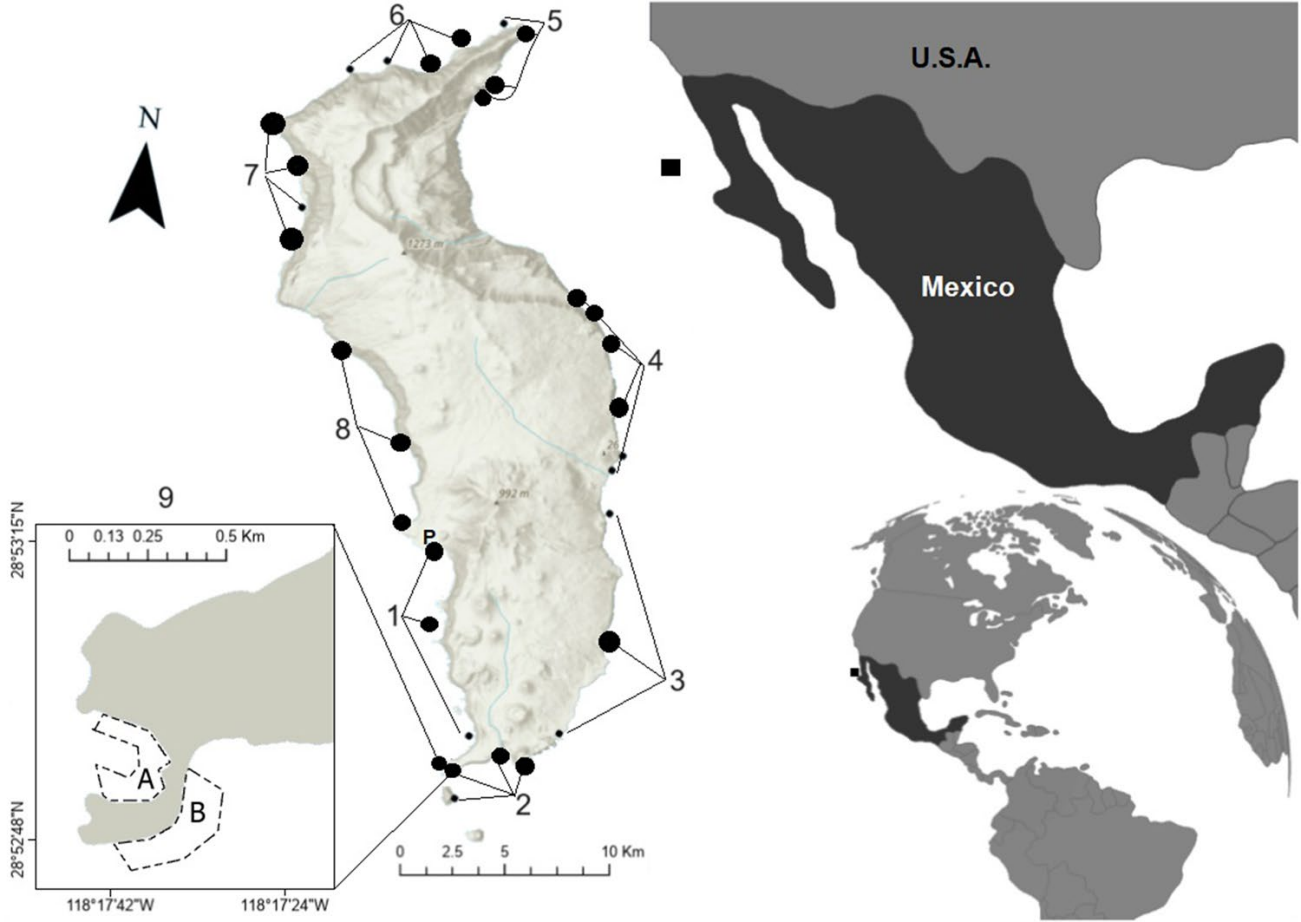
Geographic location of Guadalupe Island, Baja California, Mexico, and surveyed sites. Black dots represent monitoring sites. Small dots were monitored only in 2020, and wide dots were monitored in 2020 and 2021. Numbers indicate grouped site locations based on the cooperative official fishing polygons: Fished (locations 1–8) and marine reserves (location 9). The Inset map indicates marine reserve “Plancha” (A) and marine reserve “Gaviota” (B). P indicates port location. The map was created with ArcGis V. 10.7.1.

Guadalupe is an oceanic island of volcanic origin located within the Mexican Pacific, 241 km off the western coast of the Baja California Peninsula (Fig. 1). Guadalupe Island is located in a transition zone in the Warm Temperate Northeast Pacific marine province, between the Southern California Bight and the Magdalena Transition ecoregions^31^. This particular location results in high ecosystem complexity and endemism. Guadalupe Island has the highest conservation status by the Mexican Government of Biosphere Reserve since 2005^31^.

### Marine reserve establishment and monitoring

On July 1st, 2019, the first MR named “Plancha”, was established by the fishing cooperative. Plancha MR covers an area of 9707 m^2^ and was designated based on the ecological knowledge of the local fishing community^32^. The selection of this site was primarily influenced by its natural protection as a bay and the abundant presence of macroalgae, the main food source for abalone. Following this, the second MR, “Gaviota”, was established on July 1st, 2020, encompassing an area of 10,885 m^2^. Gaviota MR, located at the wave-protected southern tip of Guadalupe Island, is recognized by local fishers for its importance as a green abalone reproductive aggregation site, further emphasizing the significance of establishing this second MR.

**Figure 2:**
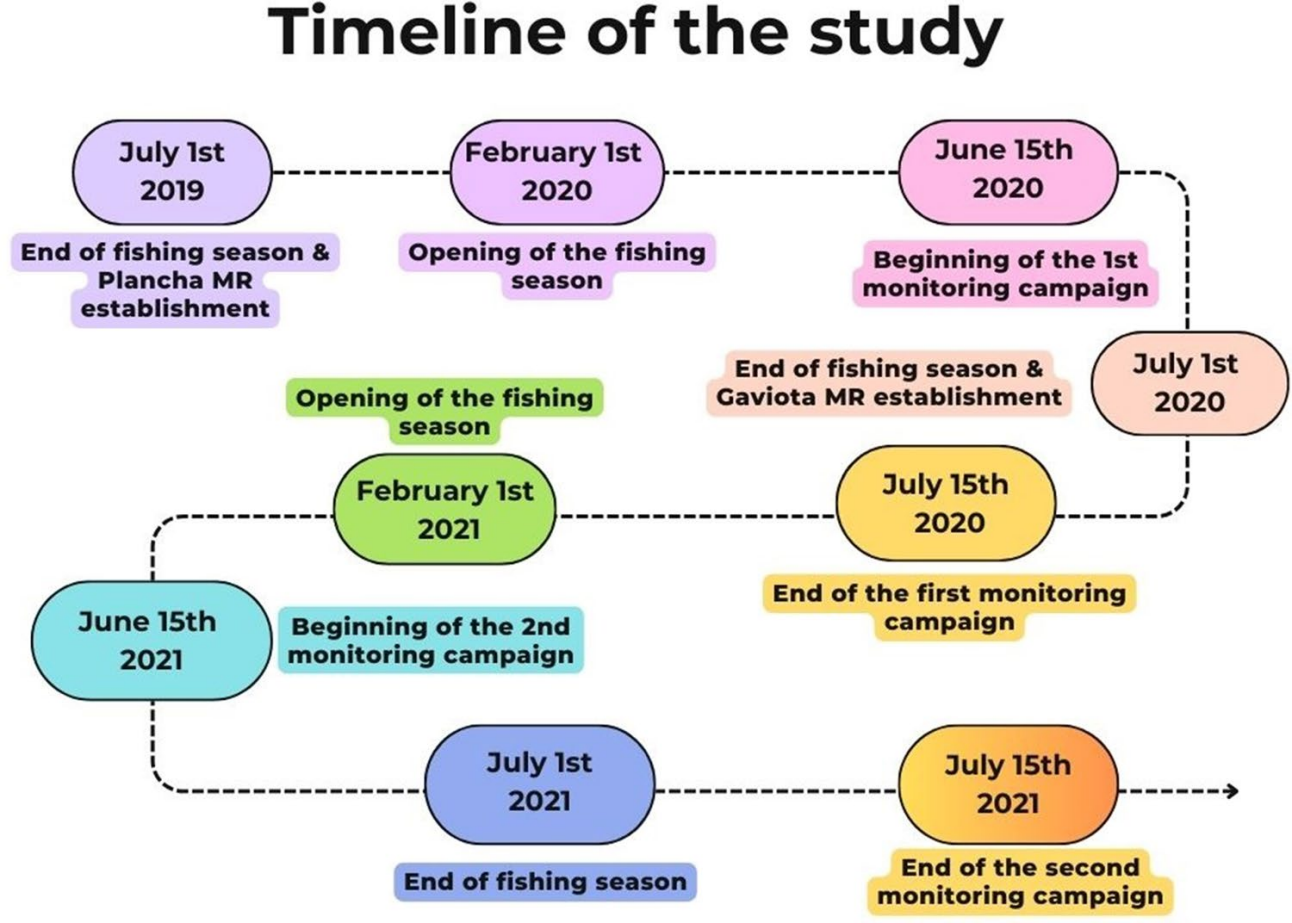
Timeline of the study development at Guadalupe Island. The abalone fishery is open from February 1st to June 30th, then has a complete closure from July 1st to January 31st (Mexican fishing zone 1 in 2021).

We conducted two monitoring campaigns to evaluate Guadalupe Island’s green abalone populations at 32 sites around the island, including 30 fishing sites and the 2 MRs (Gaviota is considered a fishing site in 2020 and a MR in 2021). The first monitoring campaign took place from June 15th to July 15th, 2020, 1 year after the establishment of Plancha MR and just before the closure of Gaviota MR. The second monitoring campaign occurred from June 15th to July 15th, 2021, marking 2 years since the establishment of Plancha MR and 1 year since the establishment of Gaviota MR. Both monitoring campaigns coincided with the end of the fishing season, following the Mexican fishery zone 1 (2021: Februrary 1st–June 30th) (Fig. 2).

In the 2020 monitoring campaign, we deployed 94 transects at 31 sites, which included 2 transects inside the Plancha MR and 92 in fishing sites. For the 2021 campaign, we deployed 92 transects at 22 sites, with 7 transects inside and 85 outside the MRs. To facilitate the interpretation of our results, we grouped the 32 sites into 9 site locations: fishing sites numbered from 1 to 8, and MRs represented by location 9. The grouping was based on the official fishing polygons defined by the cooperative for the rotational fishery (Fig. 1). The rationale behind this grouping was to align the study with the cooperative's specific interests in assessing their fishery management within the rotational polygons. Therefore, the grouping was not driven by any additional criteria but rather by the cooperative's desire to obtain data relevant to their rotational polygon-based fishery management. Our belt transects covered areas of 60 m^2^ each and were placed haphazardly on the rocky seafloor at depths ranging from 1 to 20 m, corresponding to the locations of the local abalone fishery.

During these surveys, the best way that the visual sample and benthic habitat allowed, we recorded every green abalone with the total shell length in cm, measured by hand using the scale marked on the data table. We converted the data to mm. We also logged their location on the transect. With these data, we calculated the population parameters: proportion of individuals bigger than 150 mm (minimum harvest size), estimated body weight, density, total biomass, aggregations, and potential egg production.

Within each transect, we calculated the proportion (%) of abalone exceeding the minimum harvest size of 150 mm by tallying the number and dividing it by the total number of observed abalone.

1 $${\text{Proportion}}\;\left( \% \right) = \left( {\frac{{Abalone > 150\,{\text{mm}}}}{Total\;number\;of\;observed\;abalone}} \right) \times 100$$

We recorded aggregations at each transect as the number of groups of two or more abalone with no more than 1.5 m between two individuals, a critical distance for fertilization success for abalone^33^. We then obtained the number of individuals aggregated per transect.

We calculated the density (*D*, individuals·m^2^) by dividing the number of abalone counted by the area. We converted abalone shell length to body weight (*W*) modifying the formula reported in^34^ with a local, species-specific estimate of length–weight (R^2^ = 0.953, *P* < 0.05):

2 $$W = 2.24 \times 10^{ - 5} \,{\text{L}}^{3.36} ,$$

Then we obtained the total biomass (TB) per transect.

3 $${\text{TB}} = \sum {\text{individual biomass values}}$$

We determined the eggs produced per mature female (*E*) based on^35^:

4 $$E = 0.001\,L^{1.5382}$$

where L is the green abalone shell length in mm.

We then calculated the potential egg production using adult abalones (> 49 mm as recommended for red abalone *H. rufescens* in^36^ with the mean weights and densities per transect. Comparisons of egg production were then made based on protection (MRs vs. fished sites).

We also estimated the potential egg production per m^2^:

5 $$P_{s} = {{\left( {E\left( {O_{{ > 49\,{\text{mm}}}} } \right)S} \right)} \mathord{\left/ {\vphantom {{\left( {E\left( {O_{{ > 49\,{\text{mm}}}} } \right)S} \right)} {\text{A}}}} \right. \kern-0pt} {\text{A}}},$$

where *P*_*s*_ is the potential egg production per m^2^, *E* is the eggs produced per mature female green abalone (obtained in Eq. 4), *O*_>49 mm_ is all counted adult organisms (> 49 mm), *S* is the sex ratio (0.5), and A is the monitoring area (m^2^). We report egg production in millions of eggs and use eggs·m^2^ for the discussion.

### Statistical analyses

For the analyses, we used grouped site locations. Then, we analyzed the data at the transect level (N). In this approach, each transect was treated as an individual data point. We calculated the mean value for all the transects within each grouped site location. We tested for differences in abalone shell length, densities, total biomass, aggregations, egg production, and proportion of individuals > 150 mm among the categorical factor locations. Due to violations of homoscedasticity and variance assumptions in our data, we resorted to Kruskal–Wallis (K–W) tests for each year separately followed by Dunn tests. We analyzed the effect of the protection of MRs in the abalone biological variables against fished sites per year with Mann–Whitney U tests (M–W). Lastly, we analyzed changes inside each MR using M–W tests with both years as independent variables against the biological variables. We conducted the statistical analyses with R v.4.1.1 (R Core Team 2020) and JMP V.16 (Statistical Discovery LLC).

## Results

The total monitored area during the 2 years was 11,160 m^2^ from 186 transects of 60 m^2^ each. In the first monitoring campaign in 2020, we monitored 5640 m^2^, and sampled 1220 green abalone equal to a mean density (± SE) of 0.21 ± 0.02 ind·m^2^ with a mean size of 133.91 ± 1.46 mm and a mean estimated weight of 405.90 ± 11.32 g. For the second campaign in 2021, we monitored 5520 m^2^, and sampled 1107 green abalone equal to a mean density of 0.20 ± 0.02 ind·m^2^ with a mean size of 138 ± 0.75 mm and a mean weight of 421.73 ± 5.99 g.

### Effects of grouped site location

#### 2020 Monitoring campaign

**Figure 3:**
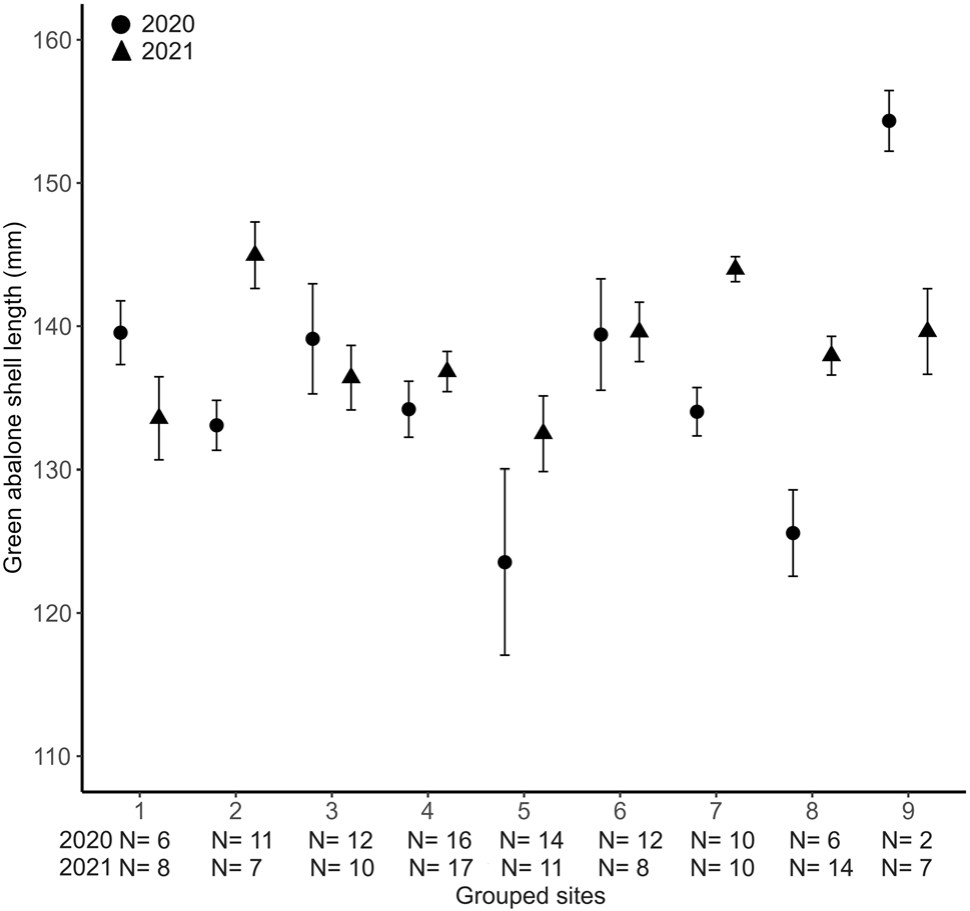
Mean green abalone shell length (mm) per transect by grouped site location. Fished groups (1–8); Marine reserves (9). The 2020 monitoring campaign is represented with circles, K-W, χ^2^ = 19.63, df = 8, P = 0.0119. The 2021 monitoring campaign is represented with triangles, K-W, χ^2^ = 16.65, df = 8, P = 0.0004. N are monitoring transects of 60 m^2^ by year. Vertical lines denote ± SE.

**Figure 4:**
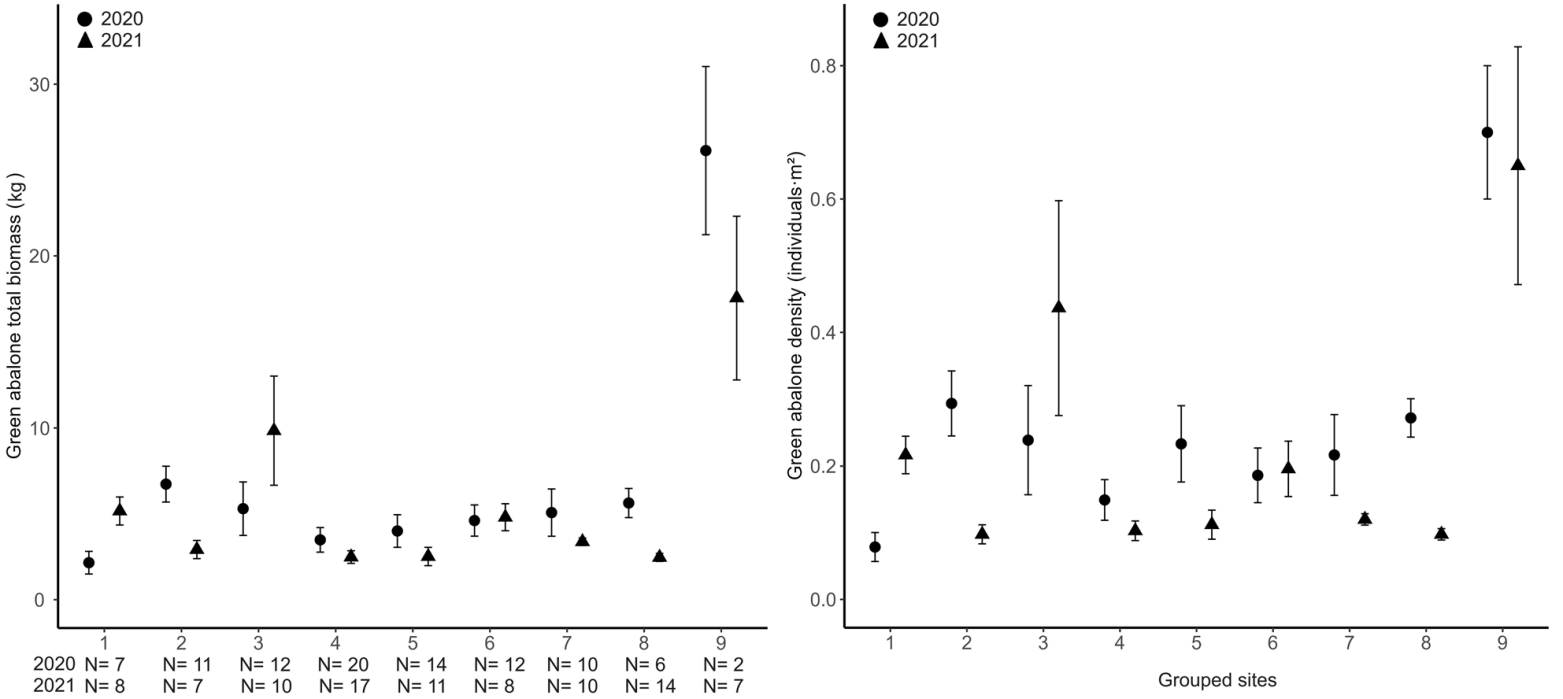
Mean green abalone total biomass (kg) and density (individuals·m^2^) per transect by site locations. Fished groups (1–8); Marine reserves (9) in 2020 Plancha MR and in 2021 Plancha MR + Gaviota MR. The monitoring campaign 2020 is represented with circles and 2021 triangles. Total biomass 2020 (χ^2^ = 16.65, df = 8, P = 0.0340), 2021 (χ^2^ = 51.08, df = 8, P < 0.0001). Density 2020 (χ^2^ = 17.63, df = 8, P = 0.0242), 2021 (χ^2^ = 49.74, df = 8, P < 0.001). N are monitoring transects of 60 m^2^ by year. Vertical lines denote ± SE.

**Figure 5:**
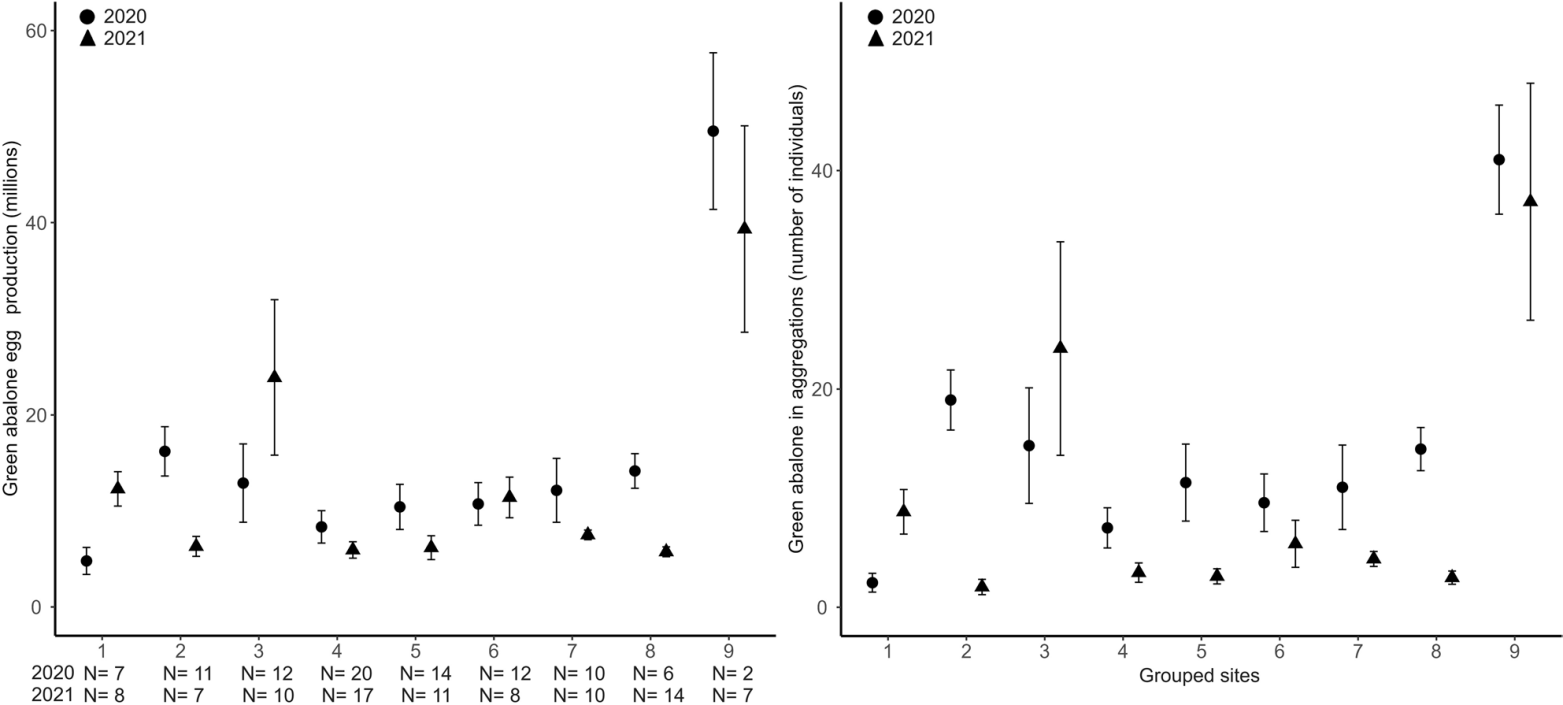
Mean green abalone egg production (millions) and aggregation (number of individuals) per transect by grouped site location. Fished groups (1–8); Marine reserves (9) in 2020 Plancha MR and 2021 Plancha MR + Gaviota MR. The monitoring campaign 2020 is represented with circles and 2021 triangles. Egg production 2020 (χ^2^ = 16.80, df = 8, P = 0.0323), 2021 (χ^2^ = 51.46, df = 8, P < 0.001). Aggregation 2020 (χ^2^ = 21.99, df = 8, P = 0.0049), 2021 (χ^2^ = 42.57, df = 8, P < 0.0001). N are monitoring transects of 60 m^2^ by year. Vertical lines denote ± SE.

We evaluated the effect of grouped site location around Guadalupe Island for the 2020 monitoring campaign. There was an effect on abalone shell length (Fig. 3; χ^2^ = 19.63, df = 8, *P* = 0.0119), total biomass (Fig. 4, χ^2^ = 16.65, df = 8, *P* = 0.0340), density (Fig. 4, χ^2^ = 17.63, df = 8, *P* = 0.0242), egg production (Fig. 5, χ^2^ = 16.80, df = 8, *P* = 0.0323), and aggregation (Fig. 5, χ^2^ = 21.99, df = 8, *P* = 0.0049). This effect was not significant for the proportion of abalone > 150 mm (χ^2^ = 11.12, df = 8, *P* = 0.195). Nonetheless, Dunn tests indicated that Plancha MR (location 9) differed from locations 4, 7, 8, 5, 1, and 6 in the proportion of abalone > 150 mm. The results from Dunn tests are in Appendix Table S2.

#### 2021 Monitoring campaign

We evaluated the effect of grouped site location around Guadalupe Island for the 2021 monitoring campaign. There was an effect on abalone shell length (Fig. 3; χ^2^ = 16.65, df = 8, *P* = 0.0004), total biomass (Fig. 4, χ^2^ = 51.08, df = 8, *P* < 0.0001), density (Fig. 4, χ^2^ = 49.74, df = 8, *P* < 0.001), egg production (Fig. 5, χ^2^ = 51.46, df = 8, *P* < 0.001), aggregation (Fig. 5, χ^2^ = 42.57, df = 8, *P* < 0.0001), and proportion of abalone > 150 mm (χ^2^ = 44.11, df = 8, *P* < 0.0001). The results from Dunn tests are in Appendix Table S2.

### Marine reserves versus fished sites

#### Marine reserve in 2020

**Figure 6:**
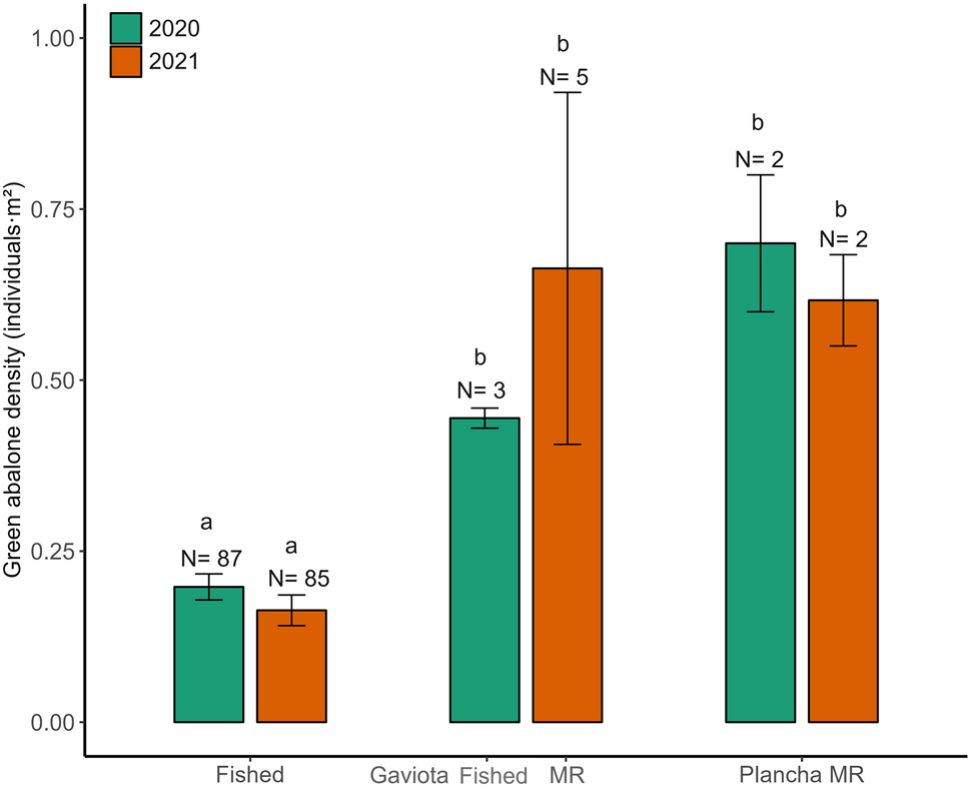
Mean green abalone density (individuals·m^2^) in fished sites and marine reserves (MR) in 2020 (green) and 2021 (orange). Gaviota was open to the fishery in 2020 and a MR in 2021. Plancha was closed to fishery in 2020 and 2021. N = monitoring transects of 60 m^2^. Fished sites were different than the MR in 2020 (χ^2^ = 10.54, df = 2, P = 0.0051), and 2021 (χ^2^ = 17.26, df = 2, P = 0.0002). There were no differences inside fishing locations or MRs across the years. Vertical lines denote ± SE.

The first MR Plancha was established by the local cooperative on July 1st, 2019, a year before the first monitoring campaign in 2020 (Fig. 2). For these results, we used transects in Plancha MR (N = 2) versus the fished sites (N = 94) for all parameters except size (N = 88) because six transects had no abalone. Green abalone mean (± SE) shell length appears to be larger inside the MR (U = 169, *P* = 0.0338; MR = 154.34 ± 2.12; fished = 133.56 ± 1.46 mm), and with a greater mean proportion of abalone above the minimum harvest size (U = 175, *P* = 0.0188; MR = 67.36 ± 3.57; fished = 13.70 ± 1.90%). Moreover, the mean density was 3.2 times higher in the MR compared with fished sites (Fig. 6, U = 181.5, *P* = 0.0241; MR = 0.7 ± 0.1; fished = 0.21 ± 0.02 ind·m^2^). These differences made the biomass within the MR 5.7 times higher than the fished sites (U = 187, *P* = 0.0165; MR = 26.13 ± 3.46; fished = 4.55 ± 0.39 kg). We also documented higher aggregation (U = 174, *P* = 0.0233; MR = 41.00 ± 5.00; fished = 10.85 ± 1.20 individuals), and egg production (U = 186, *P* = 0.0177; MR = 41.60 ± 8.46; fished = 11.31 ± 1.19 millions) (Table 1).

**Table 1:** Mann–Whitney tests of protection (fished vs. reserve) effect on green abalone size, proportion of abalone larger than 150 mm, total biomass, density, aggregation, and egg production.

2020	Mean Plancha MR ± SE	Mean fished locations ± SE	U	Z	df	*P*
Size (mm)	154.34 ± 2.12	133.45 ± 1.46	169	2.12	1	0.0338*
Proportion > 150 mm (%)	63.36 ± 3.57	13.70 ± 1.90	175	2.35	1	0.0188*
Total biomass (kg)	26.13 ± 4.90	4.55 ± 0.39	187	2.40	1	0.0159*
Density (ind·m^2^)	0.7 ± 0.1	0.21 ± 0.02	181.5	2.26	1	0.0233*
Aggregation (ind)	41.00 ± 5.00	10.85 ± 1.20	174	2.27	1	0.0233*
Eggs (millions)	41.60 ± 8.46	11.00 ± 0.96	186	2.37	1	0.0171*

Data for the 2020 monitoring campaign: N = 94 transects.N in Plancha MR location (9) = 2; N in fished locations (1–8) = 92 for all biological parameters except for the size (six transects without abalone) N = 86. *df*  degrees of freedom, *ind* individuals. 150 mm is the minimum harvest size for green abalone. Aggregation is the mean number of individuals aggregated by transects. * indicates statistical significance.

#### Marine reserves in 2021

The second MR, Gaviota, was established on July 1st, 2020. For the 2021 monitoring campaign, Plancha MR had been closed to abalone fishing for 2 years and Gaviota MR for 1 year (Fig. 2). For these results, we used transects in both MRs (N = 7) versus the fished sites (N = 85). Again, in the MRs we found a higher mean (± SE) abalone proportion above the harvest size (U = 557, *P* < 0.0001; MRs = 14.70 ± 3.71; fished = 2.94 ± 0.64%), density (Fig. 6, U = 606.5, *P* < 0.0001; MRs = 0.65 ± 0.08; fished = 0.18 ± 0.04 ind·m^2^), biomass (Fig. 4; U = 606, *P* < 0.0001; MRs = 17.55 ± 4.40; fished = 3.96 ± 0.46 kg), aggregation (U = 615, *P* < 0.0001; MRs = 37.14 ± 10.86; fished = 6.29 ± 1.36 ind) and potential egg production (U = 606, *P* < 0.0001; MRs = 39.33 ± 10.75; fished = 10.31 ± 1.96 millions). This time, MRs had no effect on mean abalone shell length compared with fished sites (U = 373, *P* = 0.4838; MRs = 139.63 ± 2.99; fished = 136.75 ± 1.18 mm) (Table 2).

**Table 2:** Mann–Whitney tests of protection (fished vs. reserve) effect on green abalone size, proportion of abalone larger than 150 mm, total biomass, density, aggregations, and egg production.

2021 monitoring campaign	Mean Plancha MR + Gaviota MR ± SE	Mean fished locations ± SE	U	Z	df	*P*
Size (mm)	139.63 ± 2.99	137.87 ± 0.78	373	0.69	1	0.4885
Proportion > 150 mm (%)	14.70 ± 3.71	2.94 ± 0.64	557.5	4.24	1	< 0.0001*
Total biomass (kg)	17.55 ± 4.80	3.96 ± 0.47	606	4.12	1	< 0.0001*
Density (ind·m^2^)	0.65 ± 0.18	0.16 ± 0.02	606.5	4.14	1	< 0.0001*
Aggregation (ind)	37.14 ± 10.86	6.29 ± 1.36	615	4.19	1	< 0.0001*
Eggs (millions)	39.33 ± 10.75	9.37 ± 1.16	606	4.12	1	< 0.0001*

Data for the 2021 monitoring campaign: N = 92 transects. N in MRs location (9) = 7; N in fished locations (1–8) = 85 for all biological parameters. *df* degrees of freedom, *ind* individuals. 150 mm is the minimum harvest size for green abalone. Aggregation is the mean number of individuals aggregated by transects. * indicates statistical significance.

After 1 year of being closed to harvesting, Wilcoxon tests indicate no differences among biological parameters within Gaviota MR. Nonetheless, the mean (± SE) density increased by 50.00% (before = 0.44 ± 0.02, N = 3; after = 0.66 ± 0.26 ind·m^2^; U = 15, *P* = 0.7656). There was also a 6.62% increase in shell length (before = 128.26 ± 0.61; after = 136.76 ± 2.96 mm; U = 9, *P* = 0.2330) which resulted in an 82.67% increase in biomass (before = 9.29 ± 0.39; after = 16.97 ± 6.14 kg; U = 11, *P* = 0.5510), 66.36% in egg production (before = 23.51 ± 0.83; after = 39.11 ± 15.56; U = 15, *P* = 0.7656), 94.09% increase in proportion of abalone above the harvest size (before = 4.91 ± 3.13; after = 9.53 ± 1.48%; U = 9, *P* = 0.233), and 51.02% in aggregation (before = 25.00 ± 1.73; after = 37.80 ± 15.69 individuals; U = 14.5, *P* = 0.8808) (Table 3).

**Table 3:** Mann–Whitney tests of the site Gaviota before closure to the fishery in the 2020 monitoring campaign (N = 3) versus Gaviota 1 year after the marine reserve was established in the 2021 campaign (N = 5).

Source	Mean Gaviota fished 2020 ± SE	Mean Gaviota MR 2021 ± SE	U	Z	df	*P*
Size (mm)	128.25 ± 0.61	139.17 ± 15.10	9	− 1.19	1	0.233
Proportion > 150 mm (%)	4.91 ± 3.13	9.53 ± 1.48	9	− 1.19	1	0.233
Density (ind·m^2^)	0.44 ± 0.02	0.66 ± 0.26	15	0.30	1	0.766
Total biomass (kg)	9.29 ± 0.48	16.97 ± 6.87	11	− 0.60	1	0.456
Eggs (millions)	23.51 ± 0.83	39.11 ± 15.56	15	0.30	1	0.766
Aggregation (ind)	25.00 ± 1.73	37.80 ± 15.69	14.5	0.15	1	0.881

*df* degrees of freedom, *ind* individuals. 150 mm is the minimum harvest size for green abalone. Aggregation is the mean number of individuals aggregated by transects.

After 2 years of closure, demographic and biological parameters at Plancha MR remained relatively constant; Wilcoxon tests indicated no differences among population parameters. Nevertheless, we recorded a small decrease of 11.43% in the mean density (2020 = 0.70 ± 0.1, N = 2; 2021 = 0.62 ± 0.07 ind·m^2^, N = 2; U = 4, *P* = 0.6985). Also, the shell length had a 4.89% decrease (2020 = 154.34 ± 2.12; 2021 = 146.79 ± 5.33 mm; U = 3, *P* = 0.2453). This combination resulted in a 27.25% decrease in biomass (2020 = 26.13 ± 3.46; 2021 = 19.01 ± 0.40 kg; U = 3, *P* = 0.1213), 19.48% in egg production (2020 = 49.53 ± 8.16; 2021 = 39.88 ± 2.30 millions; U = 4, *P* = 0.4386). The proportion of abalone above harvest size also decreased by 58.97% (2020 = 67.36 ± 3.47; 2021 = 27.64 ± 5.69; U = 3, *P* = 0.245), and aggregation by 13.42% (2020 = 41 ± 5; 2021 = 35.5 ± 3.5; U = 4, *P* = 0.6985) (Table 4).

**Table 4:** Mann–Whitney tests of the site Plancha 1 year after closure to fishery in the 2020 monitoring campaign (N = 2) versus Plancha 2 years after being closed in the 2021 campaign (N = 2).

Source	Mean Plancha MR 2020 ± SE	Mean Plancha MR 2021 ± SE	U	Z	df	*P*
Size (mm)	154.34 ± 2.12	146.79 ± 5.33	3	− 1.16	1	0.245
Proportion > 150 mm (%)	67.36 ± 3.47	27.64 ± 5.69	3	− 1.16	1	0.123
Density (ind·m^2^)	0.70 ± 0.1	0.62 ± 0.07	4	− 0.39	1	0.699
Total biomass (kg)	26.13 ± 4.90	19.01 ± 0.57	3	− 1.16	1	0.245
Aggregation (ind)	41.00 ± 5.00	35.50 ± 3.50	4	− 0.39	1	0.699
Eggs (millions)	49.53 ± 8.16	39.88 ± 2.30	4	− 0.38	1	0.699

*df* degrees of freedom, *ind* individuals. 150 mm is the minimum harvest size for green abalone. Aggregation is the mean number of individuals aggregated by transects.

## Discussion

We assessed the green abalone population and analyzed the short-term effects of two marine reserves (MRs) in the Northeastern Pacific. Our study is the first to report data on the green abalone population around Guadalupe Island. We focus on the density, biomass, aggregation, and egg production of green abalone as short-term positive effects of MRs. This work validates the relevance of protection and highlights the need for more studies to unravel complex population status, biophysical dynamics, and social-ecological attributes of this fishery.

Regarding the MRs, the cooperative established Plancha MR 1 year before our study (July 1st, 2019), followed by the establishment of Gaviota MR in the middle of our study (July 1st, 2020). Our results reveal an average density three times higher in both MRs than in fished sites. During the first monitoring campaign, Plancha MR exhibited the highest mean (± SE) density (0.7 ± 0.1 ind·m^2^) and biomass (26.13 ± 3.46 kg) per transect in the survey, with a small decrease in 2021 (0.62 ± 0.06 ind·m^2^; 19.01 ± 0.40 kg). The closure of the Gaviota MR caused a 50% increase in mean density (before = 0.44 ± 0.02; after = 0.66 ± 0.26 ind·m^2^), which resulted in an 88.67% increase in mean biomass (before = 9.29 ± 0.39; after = 16.97 ± 6.14 kg). However, it is essential to exercise caution in attributing these observed effects solely to MR protection. The response may be influenced by the spatial heterogeneity of the benthic environment and other confounding factors^37^. Additionally, the limited number of transects within the MRs could result in wider standard errors. Nonetheless, this does not imply that MRs are ineffective, but that the positive effects observed in our study with green abalone, and other studies with fishes^11,38^, echinoderms^39,40^, and gastropods^5^, including abalone^4,36,41,42^ should be interpreted with caution.

While our primary objective aimed to cover multiple locations across Guadalupe Island to better characterize the green abalone population, we acknowledge that the unequal distribution in sampling efforts could have implications. The uneven number of transects among different protection areas can lead to variations in the data. This variability could be a result of both natural differences in the population and the limited sample size. Also, the standard error may appear abnormally low in cases where the sample size is small. Lastly, the disparities in sampling effort might affect the representativeness of our observations. Some areas with fewer transects may not fully capture the diversity and dynamics of the green abalone population.

Our data suggests that abalone aggregations in MRs were 4–6 times higher than in fished sites (2020, Plancha MR = 41.00 ± 5.00; fished = 10.85 ± 1.20 ind; 2021, Plancha + Gaviota MR = 37.14 ± 10.86; fished = 6.29 ± 1.36 individuals per transect). Given abalone are broadcast spawners, bigger and more aggregated individuals can enhance fertilization rates potentially resulting in a positive effect on the population. This has been reported for green abalone by Parnell et al.^40^ as long term effects of Californian MRs. Yet, to our knowledge, this is the first report in the region concerning short-term changes in green abalone aggregations after only 2 years of fishing closures.

**Figure 7:**
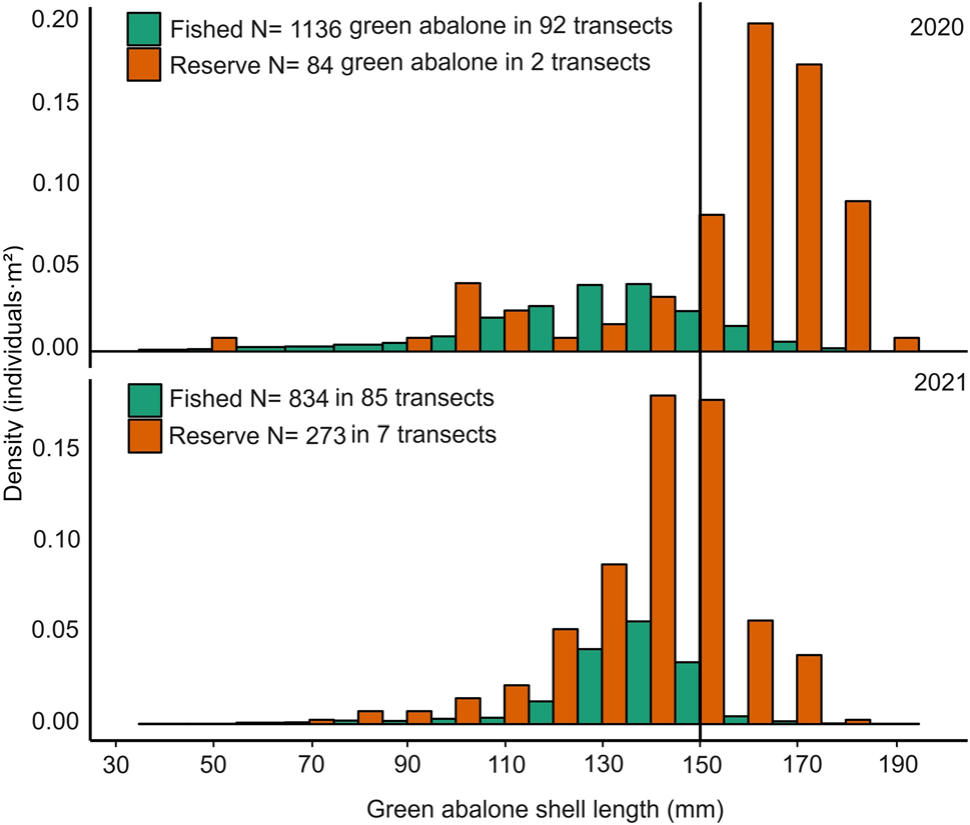
Overlapping size (mm) structure and density (individuals·m^2^) of green abalone in marine reserve (orange) and fished (green) sites for the 2020 and 2021 monitoring campaigns. N = abalone observations. In the 2020 monitoring campaign, we deployed 94 transects in fished sites and 2 in the marine reserve Plancha. In 2021, we deployed 85 transects in fished sites and 7 in Gaviota and Plancha marine reserves. The black line indicates the minimum harvest size (150 mm).

Short-term effects of MRs on abalone size have been previously documented^30^. However, abalone are known for their slow growth, which means that observing a noticeable change in shell length within 1–2 years is generally considered a short timeframe. Hence, one potential effect of the MRs is the increase in the number of individuals that exceed the minimum harvest size, allowing them to "escape" the fishery (Fig. 7). This can lead to a higher proportion of large abalone inside the MR, as observed in this study (2020, Plancha MR = 67.36 ± 3.57; fished = 13.70 ± 1.90%; 2021, Plancha + Gaviota MR = 14.70 ± 3.71; fished = 2.94 ± 0.64%). The increases in abalone size and aggregations inside reserves combined with a higher biomass could enhance egg production.

On average, both MRs had 4.2 times more biomass per transect than the fished areas. Plancha MR in 2020 had 5.7 times (inside = 26.13 ± 4.90; outside = 4.55 ± 0.39 kg), and both MRs 4.4 times in 2021 (inside = 17.55 ± 4.80; outside = 3.96 ± 0.47 kg). Our results agree with the previously documented Californian MRs, implying that there are stronger responses to biomass metrics than density^43–45^. Also, as suggested by Lester et al.^5^, biomass responds faster than abundance to reserve protection, on average 4–5 times, while densities are only 2–3 times, as observed in this study. Thus, the combination of larger and more abundant animals should result in a higher egg production^46^.

The establishment of MRs at Guadalupe Island suggests an average egg production 4.1 times higher than the fished sites (inside = 683,662 ± 43,583; outside = 166,522 ± 92,828 eggs·m^2^). This result agrees with^36^, who reported higher potential egg production in Californian MRs than in fished sites for abalone. Also, abalone are more likely to be found in higher aggregations at protected areas relative to exploited sites^40^, as documented on this study. Additionally, the combined responses of abalone densities and size structure to protection can increase genetic diversity and reproductive output and sustain recruitment in areas surrounding the MRs through a spillover effect^4,35,47^. For example, at Natividad Island, Baja California Sur, green abalone has a larval dispersal estimation of ⁓ 300 m^4^. This means that sites on the southern tip of Guadalupe Island could receive spillover from the reserves and need to be considered in future research. Nonetheless, due to the short-term effects of this study and the cryptic behavior of juvenile abalone, the probability of observing this effect is low. It is also important to consider that fishing mortality directly impacts the reproductive potential, and a MR is one of the few management scenarios that enhances resilience^48^.

It has been documented that spatial depletion occurs in abalone fisheries, with the fishing grounds closest to the port being depleted first, removing virtually all the available stock in those areas^49,50^. In the first monitoring campaign in 2020, we observed similar effects as location 1, the closest to port, presented the lowest mean density (0.08 ± 0.02 ind·m^2^) and biomass (2.16 ± 0.66 kg) per transect. The following effects were documented at location 4, at the island wave-protected area (Fig. 1). At these sites, fishing pressure is higher throughout the season than in the exposed part of the island. We acknowledge the importance of considering variations of exploited areas in our study, but the primary objective was to assess the population instead of the fishery.

Interestingly, we found higher abalone densities in Guadalupe Island than in most of the Northeastern Pacific, except for Van Damme State Park, California (Appendix Table S1). Such high population densities warrant studying Guadalupe Island to enhance our understanding of abalone population dynamics in remote regions. It is also imperative to consider that some sites at Guadalupe Island are at the density limit where recruitment failure is expected (i.e., 0.2–0.3 ind·m^2^) ^33^. Thus, it is of utmost importance to continue the establishment of MRs to avoid the Allee effect on the local green abalone population^26^. Still, it is important to mention that both monitoring campaigns were at the end of the fishing season (Mexican fishery zone 1 in 2021: November 30th to June 30th), and local ecological knowledge suggests that the abalone densities are around 2–3 times higher at the start of the season. These hypotheses could be related to: (1) by not fishing below 20 m, the deeper abalone can potentially supply the shallower areas with larvae and adults; (2) cryptic abalone that escape the previous year's fishery; and (3) rotational fishing management that the cooperative has used for years.

The Guadalupe Island fishing cooperative is a good example of how community management might work to increase the resilience and sustainability of local marine resources. The values of the fishing cooperative extend beyond the mere administration of marine reserves. It embodies a collaborative approach where local stakeholders actively participate in decision-making processes. This knowledge, passed down through generations, informs resource management strategies that balance conservation goals with the needs of the fishing community. By emphasizing the cooperative's role and social-ecological contributions, it becomes evident that their involvement is instrumental in achieving sustainable fisheries management and preserving species like the green abalone.

Given the results from the present study, the life history characteristics of abalone, the social-ecological system, and the unique bio-physical, oceanographic, and geographical conditions at Guadalupe Island a set of no-take zones could create a persistent network for abalone conservation^51^. The size and spacing of that network depends on the settler–recruit relationship of a particular species, adult movement, and longshore currents^52^. Abalone larvae settlement can occur within a short (< 50 m), long (> 100 m), or short and long (dual mode) distance from their parents^53^. The green abalone population at Guadalupe Island belongs to a subspecies (*H. fulgens guadalupensis*)^54,55^, suggesting that local recruitment is predominant. Therefore, present, and future MRs will likely be critical to sustaining the local abalone populations and fishery yields. Particularly considering the broader context of abalone conservation throughout the rest of the country under uncertain future climate conditions as the frequency and length of marine heatwaves and other climate impacts increase^56–58^.

Several studies document the positive effects that MRs have on abalone fishery yields^15,59,60^. Nevertheless, the placement of reserves is a critical aspect. For example, placing reserve edges in a continuous habitat may enhance spillover and thus benefit fisheries^60^. Also, if the no-take zone is too big, sessile or sedentary species rarely move out of reserves; hence they are rarely captured and provide only the benefit of larval transport^61^. Thus, to increase fishery yields, the size of the reserves is important, as the largest yields are obtained with small reserves of around 100 m wide so that the export of larvae and spillover of adults are maximized^41,59^. Finally, the goals of MRs could be enhanced if located in source^48^ or sink areas^62^. Nonetheless, payoffs of stand-alone marine reserves rarely compete with more traditional optimal management schemes^63^, such as the one applied by the fishing cooperative in this study. Still, they can be beneficial when stocks are heavily exploited^64^. Thus, the combination of MRs with good management could maintain a sustainable fishery at Guadalupe Island.

Continuing monitoring of the social-ecological system is critical to provide guidelines preventing abalone and other fished species from reaching a critical threshold beyond which recovery is virtually impossible^65^. However, we need more information to adequately evaluate the effects of establishing MRs, for example, utilizing Before-after-control-impact with a rigorous assessment of the benthic habitat^66^. Also, understanding the biological attributes of the focal species, such as ontogenetic movement with telemetry^67^, dispersal with genetic studies^53^, the population growth rate with long-term monitoring^68^, and the size of reserves relative to the home range^69^. Lastly, fisheries attributes such as the status of the fishery before implementation of reserves; fishers' behavior, and fleet dynamics before and after reserve implementations; and effective leadership and governance, including capacity for monitoring and enforcement^70^.

We acknowledge that our results cannot be fully attributed only to the MRs. For example, our observations spanned only 2 years, with the initial monitoring campaign in 2020 being essentially a single-point-in-time comparison between sites. Additionally, the selection of MR locations was not random; rather, it was based on local ecological knowledge of the cooperative^32^. These chosen locations seem favorable to green abalone biological and demographic parameters, which could be linked to specific oceanographic and habitat conditions^37^. While this non-random selection process can introduce potential biases in our findings, they are relevant factors to be considered for the success of MRs worldwide^71,72^. Furthermore, we did not provide a detailed description of the heterogeneity of the benthic habitat. This factor can significantly contribute to the observed variations in abalone parameters across different locations^73^. Nonetheless, transects were deployed only on rocky reefs where abalone fishery occurs. As highlighted by Miller et al.^74^, if MRs and control (fished) sites are initially dissimilar in habitat, larval supply, or historical conditions, comparing them can be confounded by inherent site differences.

Finally, MRs seem to be a great tool to improve abalone fishery management, but we also need to think about other technologies and adaptation strategies to cope with climate change. For example, we could boost the abalone densities and hence fertilization inside MRs with translocation efforts^75^. Another option is the development of conservation aquaculture, as sustainable mariculture systems^76^ to complement the fishery and enhance wild populations through seed restocking^77–79^. To end, there is no single panacea but a combination of co-management approaches and the development of sustainable technologies to recover the affected wild abalone populations worldwide.

### Supplementary Information

Supplementary Information.

## Data Availability

All data relevant to the study is included in the article. In addition, the datasets used and/or analyzed during the current study are available from the corresponding author upon reasonable request.

## Abbreviations

MR: Marine reserve
CONANP: Comisión Nacional de Áreas Protegidas
PROSEMAR: Proyectos y Servicios Marinos
