# Supplementary material for: Short-term effects of community-based marine reserves on green abalone, as revealed by population studies
Source: Sci Rep. 2024 Jan 10;14:955. doi: 10.1038/s41598-023-50316-9 (PMC10781752; doi:10.1038/s41598-023-50316-9)
Supplement: Supplementary file 1 — Supplementary Information. [file 41598_2023_50316_MOESM1_ESM.docx]

**Supplementary information**

| Supplementary table 1. Densities (individual·m^2^) of different abalone species on the Northeastern Pacific. When information was available, we compared inside and outside marine reserves. Locations are listed from south to north. | | | | | |
| --- | --- | --- | --- | --- | --- |
| **Authors** | **Latitude**  **longitude** | **Location** | **Protection** | **Abalone spp.** | **Density**  **(Individual m^-2^)** |
| Guzmán del Próo *et al*. (2013) ^81^ | 27.68  -114.87 | Bahía Tortugas, Baja California Sur, Mexico | Fished site | Green *H. fulgens* | 1997 - 0.12  2009 - 0.12 |
| Rossetto *et al*. (2013) ^35^ | 27.87  -115.18 | Natividad Island, Baja California Sur, Mexico | No-take marine reserves with local enforcement and fished sites | Green *H. fulgens* | Inside MR  2008 - 0.0135  2009 - 0.0062  Outside MR  2008 - 0.0159  2009 - 0.0157 |
| Smith *et al*. (2022) ^23^ | 27.87  -115.18 | Natividad Island, Baja California Sur, Mexico | No-take marine reserves with local enforcement and fished sites | Green *H. fulgens* | Inside MR  2007 – 2011 - 0.16  2012 – 2016 - 0.14  2017 – 2019 - 0.10  Outside MR  2007 – 2011 - 0.11  2012 – 2016 - 0.15  2017 – 2019 - 0.10 |
| **This study** | **29.02**  **-118.27** | **Guadalupe Island, Baja California, Mexico** | **No-take marine reserves with local enforcement and fished sites** | **Green *H. fulgens guadalupensis*** | **Inside MR**  **2020 – 2021 - 0.66**  **Outside MR**  **2020 – 2021 - 0.20** |
| Rogers-Bennett *et al*. (2002) ^36^ | 32.89  -118.48 | San Clemente Island, California, U.S.A. | Federal closure of the fishery | White *H. sorenseni* | 0.047 |
| Davis *et al*. (1996) ^82^ | 33.62  -119.33 | Chanel Islands, California, U.S.A. | Federal closure of the fishery | White *H. sorenseni* | 0.0002 |
| Rogers-Bennett *et al*. (2004) ^83^ | 33.97  -120.08 | Santa Rosa Island, California, U.S.A. | Federal closure of the fishery | Red *H. rufescens* | 2000 - 0.007  2001 - 0.001  2002 - 0.001 |
| Rogers-Bennett *et al*. (2004) ^83^ | 34.00  -119.39 | Anacapa Island, South California, U.S.A. | Federal closure of the fishery | Pink *H. corrugata* | 2000 – 2003 -0.001 |
| Rogers-Bennett *et al*. (2004) ^83^ | 34.02  -119.76 | Santa Cruz Island, California, U.S.A. | Federal closure of the fishery | Pink *H. corrugata* and red *H. rufescens* | 2000 - 0  2001 - 0  2002 - 0 |
| Rogers-Bennett *et al*. (2004) ^83^ | 39.29  -123.80 | Van Damme State Park, California, U.S.A. |  | Red *H. rufescens,* flat *H. walallensis*, and pinto *H. kamtschatkana* | 2000 - 1.10  2001 - 0.55  2002 - 0.85 |

Supplementary figure 1. Locations from the studies in supplementary table 1. Map generated with ArcGIS V. 10.7.1 (https://www.arcgis.com).


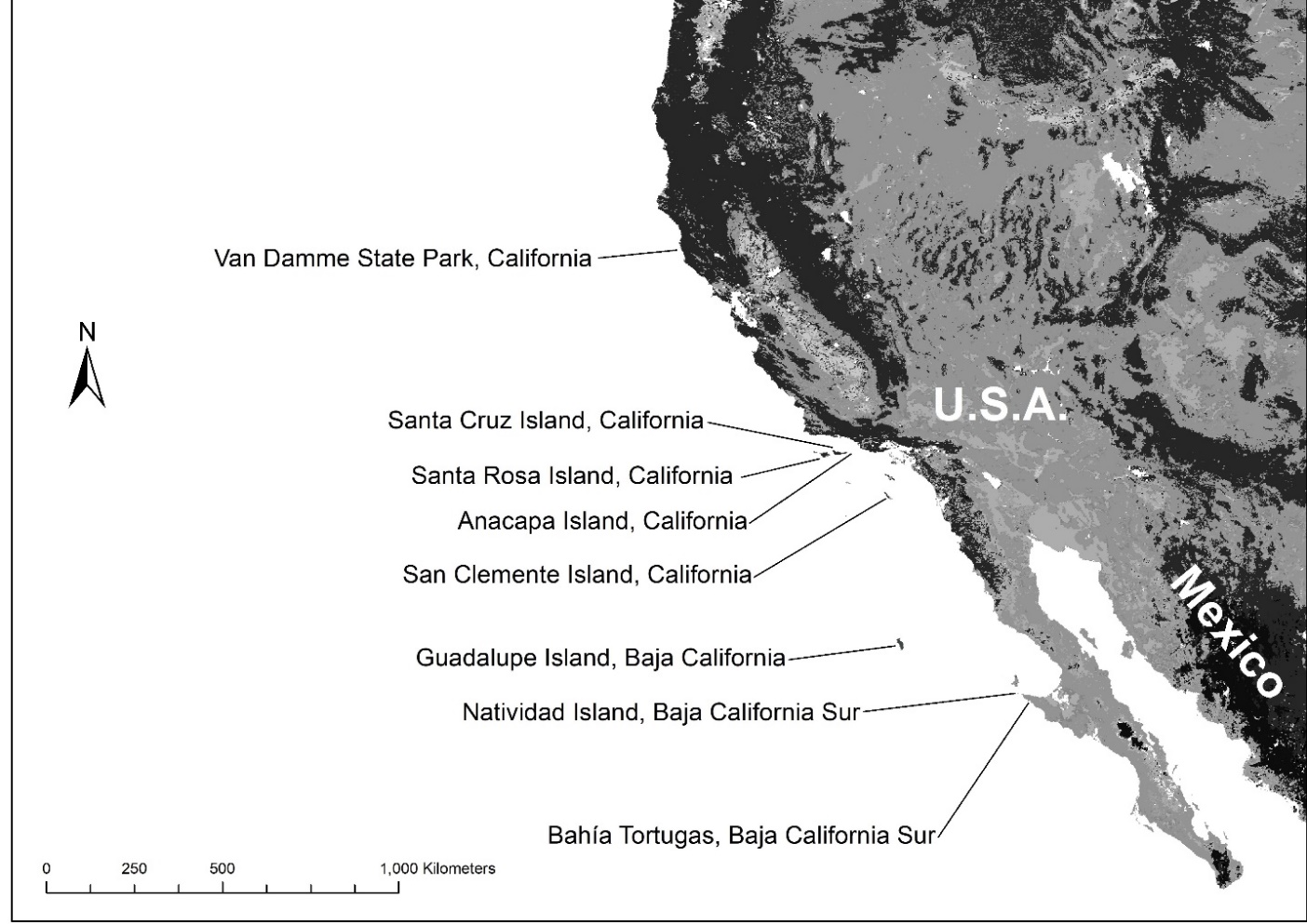


| Supplementary table 2. Results from the Dunn tests with statistically significant p-values indicating comparisons between each group of sites for each population parameter during the 2020 and 2021 monitoring campaigns. | | | | | |
| --- | --- | --- | --- | --- | --- |
| 2020 monitoring campaign Dunn test significant results for green abalone density. | | | | | |
| Level | - Level | Score Mean Difference | Standard error differences | Z | P |
| 9 | 1 | 64.1429 | 21.84951 | 2.93567 | 0.00332828 |
| 9 | 4 | 52.05 | 20.20997 | 2.57546 | 0.01001069 |
| 9 | 3 | 46.9583 | 20.81339 | 2.25616 | 0.02406061 |
| 9 | 6 | 44.75 | 20.81339 | 2.15006 | 0.03155047 |
| 9 | 7 | 42.45 | 21.10863 | 2.01103 | 0.04432229 |
| 9 | 5 | 40 | 20.59991 | 1.94176 | 0.05216616 |
| 8 | 1 | 38.2262 | 15.16112 | 2.52133 | 0.01169122 |
| 2 | 1 | 35.961 | 13.17575 | 2.72934 | 0.00634612 |
| 4 | 2 | -23.8682 | 10.22949 | -2.33327 | 0.01963398 |
| 2020 monitoring campaign Dunn test significant results for green abalone total biomass. | | | | | |
| Level | - Level | Score Mean Difference | Standard error differences | Z | P |
| 9 | 1 | 64.5357 | 21.87132 | 2.9507 | 0.00317055 |
| 9 | 4 | 54.8 | 20.23015 | 2.70883 | 0.00675209 |
| 9 | 5 | 49.7143 | 20.62048 | 2.41092 | 0.01591234 |
| 9 | 3 | 46.9583 | 20.83417 | 2.25391 | 0.02420183 |
| 9 | 7 | 44 | 21.12971 | 2.08238 | 0.03730777 |
| 9 | 6 | 43.5417 | 20.83417 | 2.08992 | 0.03662499 |
| 2 | 1 | 34.4221 | 13.1889 | 2.60993 | 0.00905608 |
| 9 | 8 | 32.8333 | 22.27267 | 1.47415 | 0.14044121 |
| 8 | 1 | 31.5357 | 15.17626 | 2.07796 | 0.03771304 |
| 4 | 2 | -24.6864 | 10.2397 | -2.41085 | 0.01591539 |
| 2020 monitoring campaign Dunn test significant results for green abalone Egg production. | | | | | |
| Level | - Level | Score Mean Difference | Standard error differences | Z | P |
| 9 | 1 | 64.4643 | 21.87061 | 2.94753 | 0.00320324 |
| 9 | 4 | 54.8 | 20.2295 | 2.70892 | 0.00675026 |
| 9 | 5 | 49.7143 | 20.61981 | 2.411 | 0.01590885 |
| 9 | 3 | 46.6667 | 20.83349 | 2.23998 | 0.02509222 |
| 9 | 7 | 44.2 | 21.12902 | 2.09191 | 0.03644657 |
| 9 | 6 | 43.7083 | 20.83349 | 2.09798 | 0.03590691 |
| 2 | 1 | 34.3506 | 13.18848 | 2.6046 | 0.00919816 |
| 4 | 2 | -24.6864 | 10.23937 | -2.41093 | 0.0159119 |
| 2020 monitoring campaign Dunn test significant results for green abalone aggregation. | | | | | |
| Level | - Level | Score Mean Difference | Standard error differences | Z | P |
| 9 | 1 | 65.125 | 20.56676 | 3.16652 | 0.00154275 |
| 9 | 4 | 49.3056 | 19.39053 | 2.54277 | 0.01099776 |
| 2 | 1 | 45.4306 | 12.64108 | 3.59388 | 0.00032579 |
| 9 | 6 | 43.4583 | 19.86938 | 2.1872 | 0.02872793 |
| 9 | 7 | 43.15 | 20.15123 | 2.14131 | 0.03224905 |
| 9 | 5 | 41.2143 | 19.66558 | 2.09576 | 0.03610349 |
| 9 | 3 | 39.75 | 19.99798 | 1.9877 | 0.04684488 |
| 8 | 1 | 38.7917 | 14.04977 | 2.76102 | 0.00576211 |
| 3 | 1 | 25.2841 | 12.08819 | 2.09164 | 0.03647073 |
| 5 | 1 | 23.8393 | 11.52997 | 2.06759 | 0.03867859 |
| 5 | 2 | -21.5198 | 11.11487 | -1.93613 | 0.05285178 |
| 7 | 2 | -23.4556 | 11.95312 | -1.9623 | 0.04972757 |
| 6 | 2 | -23.7639 | 11.47159 | -2.07154 | 0.03830836 |
| 4 | 2 | -29.6111 | 10.62063 | -2.78808 | 0.00530214 |
| 2020 monitoring campaign Dunn test significant results for green abalone proportion > 150 mm. | | | | | |
| Level | - Level | Score Mean Difference | Standard error differences | Z | P |
| 9 | 4 | 51.5833 | 18.93662 | 2.724 | 0.00644965 |
| 9 | 7 | 46.65 | 19.67951 | 2.37049 | 0.01776452 |
| 9 | 8 | 46.3333 | 20.74403 | 2.23357 | 0.02551138 |
| 9 | 5 | 42.6071 | 19.20524 | 2.21852 | 0.0265194 |
| 9 | 1 | 42.3125 | 20.08532 | 2.10664 | 0.0351488 |
| 9 | 6 | 39.625 | 19.40426 | 2.04208 | 0.0411436 |
| 4 | 3 | -20.1515 | 9.72311 | -2.07254 | 0.03821511 |
| 2021 monitoring campaign Dunn test significant results for green abalone shell length. | | | | | |
| Level | - Level | Score Mean Difference | Standard error differences | Z | P |
| 7 | 5 | 46.0364 | 11.65618 | 3.94953 | 7.83048E-05 |
| 7 | 1 | 40.9 | 12.65418 | 3.23213 | 0.001228711 |
| 2 | 1 | 39.5 | 13.80684 | 2.8609 | 0.004224403 |
| 7 | 4 | 37.1059 | 10.63164 | 3.49014 | 0.000482768 |
| 7 | 3 | 34.4 | 11.93048 | 2.88337 | 0.00393445 |
| 6 | 5 | 25.3864 | 12.39591 | 2.04796 | 0.040563919 |
| 9 | 5 | 25.3506 | 12.89834 | 1.96542 | 0.049365647 |
| 8 | 2 | -26.6071 | 12.34922 | -2.15456 | 0.031196282 |
| 8 | 7 | -28.0071 | 11.04547 | -2.53562 | 0.011224846 |
| 3 | 2 | -33 | 13.14674 | -2.51013 | 0.012068672 |
| 4 | 2 | -35.7059 | 11.9805 | -2.98033 | 0.00287938 |
| 5 | 2 | -44.6364 | 12.89834 | -3.46063 | 0.000538913 |
| 2021 monitoring campaign Dunn test significant results for green abalone density. | | | | | |
| Level | - Level | Score Mean Difference | Standard error differences | Z | P |
| 9 | 2 | 56.8571 | 14.22079 | 3.99817 | 6.38341E-05 |
| 9 | 8 | 56.5714 | 12.31556 | 4.59349 | 4.35894E-06 |
| 9 | 4 | 54.8655 | 11.94785 | 4.59209 | 4.38829E-06 |
| 9 | 5 | 54.7987 | 12.86319 | 4.26012 | 2.04317E-05 |
| 9 | 7 | 45.0714 | 13.11092 | 3.4377 | 0.000586677 |
| 3 | 2 | 43.0357 | 13.11092 | 3.28243 | 0.001029165 |
| 6 | 2 | 29.8482 | 13.76922 | 2.16775 | 0.030177713 |
| 6 | 4 | 27.8566 | 11.40665 | 2.44214 | 0.014600483 |
| 6 | 5 | 27.7898 | 12.36212 | 2.24798 | 0.024577465 |
| 7 | 1 | -25.625 | 12.6197 | -2.03056 | 0.042299649 |
| 8 | 6 | -29.5625 | 11.79125 | -2.50716 | 0.012170561 |
| 7 | 3 | -31.25 | 11.89796 | -2.6265 | 0.008626799 |
| 5 | 1 | -35.3523 | 12.36212 | -2.85972 | 0.004240152 |
| 4 | 1 | -35.4191 | 11.40665 | -3.10513 | 0.001901954 |
| 8 | 1 | -37.125 | 11.79125 | -3.14852 | 0.001640995 |
| 2 | 1 | -37.4107 | 13.76922 | -2.71698 | 0.006588059 |
| 5 | 3 | -40.9773 | 11.62441 | -3.52511 | 0.000423307 |
| 4 | 3 | -41.0441 | 10.60267 | -3.87111 | 0.000108341 |
| 8 | 3 | -42.75 | 11.01537 | -3.88094 | 0.000104054 |
| 2021 monitoring campaign Dunn test significant results for green abalone total biomass. | | | | | |
| Level | - Level | Score Mean Difference | Standard error differences | Z | P |
| 9 | 5 | 59.6818 | 12.90869 | 4.62338 | 3.77537E-06 |
| 9 | 4 | 57.6176 | 11.99012 | 4.80543 | 1.54419E-06 |
| 9 | 8 | 55.8571 | 12.35913 | 4.51951 | 6.19829E-06 |
| 9 | 2 | 49.1429 | 14.27109 | 3.44352 | 0.000574194 |
| 9 | 7 | 39.85 | 13.1573 | 3.02874 | 0.002455759 |
| 3 | 2 | 37.0929 | 13.1573 | 2.81919 | 0.004814501 |
| 6 | 5 | 34.3693 | 12.40585 | 2.77041 | 0.005598577 |
| 6 | 4 | 32.3051 | 11.447 | 2.82215 | 0.004770286 |
| 7 | 3 | -27.8 | 11.94005 | -2.3283 | 0.019896179 |
| 8 | 6 | -30.5446 | 11.83296 | -2.58132 | 0.00984233 |
| 8 | 1 | -31.3571 | 11.83296 | -2.64998 | 0.008049654 |
| 4 | 1 | -33.1176 | 11.447 | -2.89313 | 0.003814234 |
| 5 | 1 | -35.1818 | 12.40585 | -2.8359 | 0.004569675 |
| 8 | 3 | -43.8071 | 11.05434 | -3.96289 | 7.40479E-05 |
| 4 | 3 | -45.5676 | 10.64017 | -4.2826 | 1.84722E-05 |
| 5 | 3 | -47.6318 | 11.66553 | -4.08312 | 4.44351E-05 |
| 2021 monitoring campaign Dunn test significant results for green abalone egg production. | | | | | |
| Level | - Level | Score Mean Difference | Standard error differences | Z | P |
| 9 | 5 | 57.8636 | 12.90869 | 4.48253 | 7.37633E-06 |
| 9 | 8 | 56.5714 | 12.35913 | 4.5773 | 4.71016E-06 |
| 9 | 4 | 56.4412 | 11.99012 | 4.70731 | 2.51007E-06 |
| 9 | 2 | 53 | 14.27109 | 3.7138 | 0.00020417 |
| 9 | 7 | 42.25 | 13.1573 | 3.21115 | 0.001322049 |
| 3 | 2 | 40.55 | 13.1573 | 3.08194 | 0.002056563 |
| 6 | 5 | 32.3011 | 12.40585 | 2.6037 | 0.009222344 |
| 6 | 4 | 30.8787 | 11.447 | 2.69753 | 0.006985599 |
| 6 | 2 | 27.4375 | 13.81792 | 1.98565 | 0.0470722 |
| 7 | 3 | -29.8 | 11.94005 | -2.4958 | 0.012567344 |
| 8 | 6 | -31.0089 | 11.83296 | -2.62055 | 0.008778806 |
| 2 | 1 | -31.875 | 13.81792 | -2.30679 | 0.02106653 |
| 4 | 1 | -35.3162 | 11.447 | -3.08519 | 0.002034222 |
| 8 | 1 | -35.4464 | 11.83296 | -2.99557 | 0.002739324 |
| 5 | 1 | -36.7386 | 12.40585 | -2.9614 | 0.003062439 |
| 4 | 3 | -43.9912 | 10.64017 | -4.13444 | 3.55821E-05 |
| 8 | 3 | -44.1214 | 11.05434 | -3.99132 | 6.57065E-05 |
| 5 | 3 | -45.4136 | 11.66553 | -3.89298 | 9.90203E-05 |
| 2021 monitoring campaign Dunn test significant results for green abalone aggregation. | | | | | |
| Level | - Level | Score Mean Difference | Standard error differences | Z | P |
| 9 | 2 | 60.9286 | 14.31598 | 4.25598 | 2.08135E-05 |
| 9 | 8 | 54.0714 | 12.398 | 4.3613 | 1.29292E-05 |
| 9 | 5 | 53.2024 | 12.73773 | 4.17675 | 2.95704E-05 |
| 9 | 4 | 52.7563 | 12.02783 | 4.38619 | 1.15353E-05 |
| 3 | 2 | 47.3929 | 13.19868 | 3.59073 | 0.000329753 |
| 9 | 6 | 41.1494 | 12.94929 | 3.17773 | 0.001484329 |
| 9 | 7 | 39.3571 | 14.31598 | 2.74918 | 0.005974457 |
| 4 | 1 | -26.5956 | 11.48301 | -2.31608 | 0.020553901 |
| 5 | 1 | -27.0417 | 12.22459 | -2.21207 | 0.026961829 |
| 6 | 3 | -27.6136 | 11.70222 | -2.35969 | 0.018290212 |
| 8 | 1 | -27.9107 | 11.87018 | -2.35133 | 0.018706435 |
| 2 | 1 | -34.7679 | 13.86139 | -2.50825 | 0.012133079 |
| 4 | 3 | -39.2206 | 10.67364 | -3.67453 | 0.000238288 |
| 5 | 3 | -39.6667 | 11.46768 | -3.459 | 0.000542185 |
| 8 | 3 | -40.5357 | 11.08911 | -3.65545 | 0.000256731 |
| 2021 monitoring campaign Dunn test significant results for green abalone proportion > 150mm. | | | | | |
| Level | - Level | Score Mean Difference | Standard error differences | Z | P |
| 9 | 5 | 46.526 | 10.38656 | 4.47944 | 7.48391E-06 |
| 9 | 6 | 46.526 | 10.38656 | 4.47944 | 7.48391E-06 |
| 9 | 8 | 43.3214 | 9.94437 | 4.35638 | 1.32231E-05 |
| 9 | 1 | 42.1339 | 11.11814 | 3.78965 | 0.00015086 |
| 9 | 4 | 36.542 | 9.64746 | 3.78774 | 0.000152024 |
| 7 | 5 | 36.1688 | 10.38656 | 3.48227 | 0.000497182 |
| 7 | 6 | 36.1688 | 10.38656 | 3.48227 | 0.000497182 |
| 7 | 1 | 31.7768 | 11.11814 | 2.8581 | 0.004261861 |
| 9 | 2 | 30.0714 | 11.48277 | 2.61883 | 0.008823191 |
| 7 | 4 | 26.1849 | 9.64746 | 2.71417 | 0.006644206 |
| 3 | 1 | 25.1625 | 10.18995 | 2.46935 | 0.013535875 |
| 4 | 3 | -19.5706 | 8.56127 | -2.28594 | 0.022257769 |
| 8 | 3 | -26.35 | 8.89452 | -2.9625 | 0.003051518 |
| 5 | 3 | -29.5545 | 9.38629 | -3.14869 | 0.001640041 |
| 6 | 3 | -29.5545 | 9.38629 | -3.14869 | 0.001640041 |
| 8 | 7 | -32.9643 | 9.94437 | -3.31487 | 0.000916858 |

| 2020 monitoring campaign Dunn test significant results for green abalone shell length. | | | | | |
| --- | --- | --- | --- | --- | --- |
| Level | - Level | Score Mean Difference | Standard error differences | Z | P |
| 9 | 8 | 62.5 | 21.32564 | 2.93074 | 0.00338156 |
| 9 | 5 | 52.5357 | 19.7437 | 2.66088 | 0.00779367 |
| 9 | 2 | 42.9773 | 20.07742 | 2.14058 | 0.03230792 |
| 9 | 4 | 39.5556 | 19.46755 | 2.03187 | 0.04216682 |
| 9 | 7 | 39.45 | 20.23128 | 1.94995 | 0.05118208 |
| 5 | 3 | -27.2857 | 10.52343 | -2.59285 | 0.00951843 |
| 5 | 1 | -28.869 | 12.74451 | -2.26522 | 0.02349918 |
| 8 | 6 | -31.0417 | 13.05923 | -2.37699 | 0.01745456 |
| 8 | 3 | -37.25 | 13.25562 | -2.81013 | 0.00495215 |
| 8 | 1 | -38.8333 | 15.0795 | -2.57524 | 0.01001706 |
